# Nucleo-Cytoplasmic Localization Domains Regulate Krüppel-Like Factor 6 (KLF6) Protein Stability and Tumor Suppressor Function

**DOI:** 10.1371/journal.pone.0012639

**Published:** 2010-09-09

**Authors:** Estefanía Rodríguez, Nana Aburjania, Nolan M. Priedigkeit, Analisa DiFeo, John A. Martignetti

**Affiliations:** 1 Department of Genetics and Genomic Sciences, Mount Sinai School of Medicine, New York, New York, United States of America; 2 Department of Oncological Sciences, Mount Sinai School of Medicine, New York, New York, United States of America; Brunel University, United Kingdom

## Abstract

**Background:**

The tumor suppressor KLF6 and its oncogenic cytoplasmic splice variant KLF6-SV1 represent a paradigm in cancer biology in that their antagonistic cancer functions are encoded within the same gene. As a consequence of splicing, KLF6-SV1 loses both the C-terminus C_2_H_2_ three zinc finger (ZF) domain, which characterizes all KLF proteins, as well as the adjacent 5′ basic region (5BR), a putative nuclear localization signal (NLS). It has been hypothesized that this NLS is a functional domain critical to direct the distinct subcellular localization of the tumor suppressor and its splice variant.

**Methodology/Principal Findings:**

In this study, we demonstrate using EGFP fusion constructs that KLF6/KLF6-SV1 nucleo-cytoplasmic transport is not regulated by the 5′ basic region but activated by a novel NLS encoded within the ZF domain, and a nuclear export signal (NES) located in the first 16 amino acids of the shared N-terminus sequence. We demonstrate KLF6 nuclear export to be Crm1-dependent. The dysregulation of nucleo-cytoplasmic transport when disrupting the KLF6 NLS using site-directed mutagenesis showed that its integrity is necessary for appropriate protein stability. Moreover, these mutations impaired transcriptional induction of two KLF6 well-characterized target genes, E-cadherin and p21, as shown by RT-PCR and luciferase promoter assays. The addition of the ZF domain to KLF6-SV1 results in its nuclear localization and a markedly decreased half-life similar to wild type KLF6.

**Conclusions/Significance:**

We describe the domains that control KLF6 nucleo-cytoplasmic shuttling and how these domains play a role in KLF6 protein half-life and tumor suppressor function. The results begin to mechanistically explain, at least in part, the opposing functions of KLF6 and KLF6-SV1 in cancer.

## Introduction

KLF6 is a tumor suppressor gene and member of the Krüppel-like factor family of transcriptional regulators involved in development and differentiation as well as in growth signaling pathways, apoptosis, proliferation and angiogenesis [1], [2]. The tumor suppressor function of KLF6 has been widely confirmed through its loss and mutation in a number of cancers and the ability to reduce colony formation in cultured cells [1], [3], [4]–[14]. Like all members of the KLF family, KLF6 is characterized by three C-terminus C_2_H_2_ zinc fingers (ZF) that form the DNA binding domain and an N-terminus activation domain [15].

**Figure 1:**
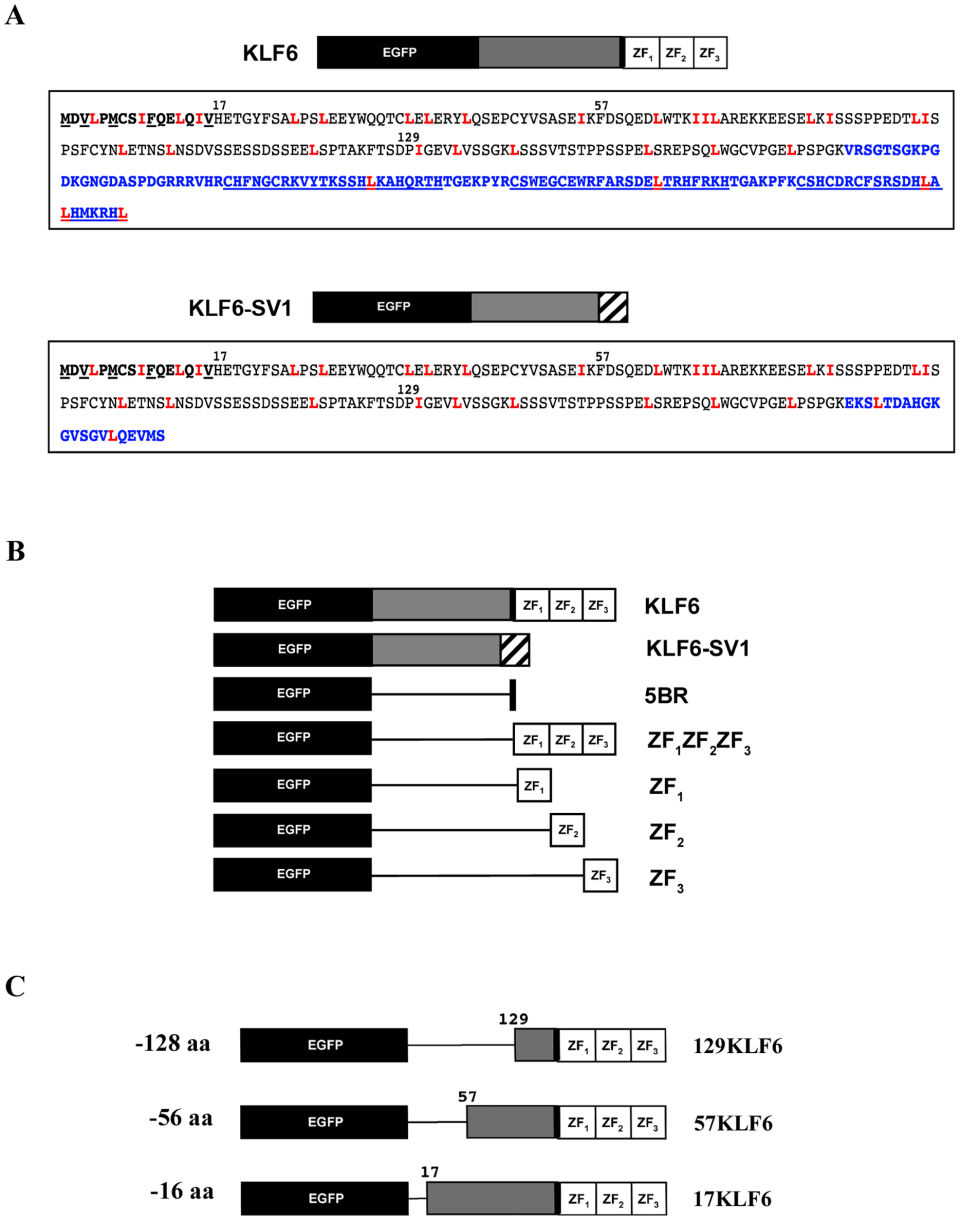
Diagram of the different EGFP constructs. A, KLF6 and KLF6-SV1 protein sequences. All Leu (L) and Ile (I) residues are highlighted in red. The 16 amino acids (aa) that form the KLF6 NES are in bold. Other hydrophobic aa within the NES are underlined. The continuous underlines in the C-terminus of the KLF6 sequence represent the three zinc fingers. The aa which differ between the two proteins are highlighted in blue. B, Diagram of the EGFP constructs used to interrogate and define the KLF6 NLS. C, Diagram of the N-terminus deletions used to identify and investigate the KLF6 NES.

Intriguingly, KLF6 is alternatively spliced into KLF6-SV1, a cytoplasmic protein that lacks the canonical KLF family DNA binding domain and the contiguous 5′ basic region (5BR), considered a putative NLS, which are both replaced by a novel C-terminal 21 amino acids (16, Figure 1A). While KLF6-SV1 appears to localize exclusively in the cytoplasm, KLF6 is present in both the nucleus and cytoplasm [16]. To date, the distinct subcellular localization differences between KLF6 and KLF6-SV1 have been attributed, respectively, to the presence or absence of the 5′ basic region. KLF6-SV1 was first shown to promote tumor growth, cancer development and metastasis in prostate cancer (PCa) [1]. Since its original identification in PCa, increased expression of this C-terminus truncated splice variant has been correlated with metastasis and poor survival not only in prostate cancer [1], [16], [17] but also in nasopharyngeal carcinoma [14], colorectal cancer [6], lung cancer [18], hepatocellular carcinoma [8], gliobastoma [4], ovarian cancer [3], head and neck squamous cell carcinoma [13] and pancreatic cancer [19]. Given the cancer-relevant and antagonistic functions of KLF6 and KLF6-SV1 it will be important to define the functionality of the putative NLS, the 5BR, as well as the role of nucleo-cytoplasmic shuttling in regulating KLF6/KLF6-SV1 function.

Regarding subcellular localization domains, the putative NLS has been shown to be functional in KLF1 and KLF4. Moreover, the KLF zinc finger domain has also been implicated in driving nuclear localization of these proteins [20]–[23]. On the other hand, only KLF5 has been demonstrated to possess a nuclear export signal (NES) [24]. In general, subcellular trafficking depends on the presence of specific functional domains within protein sequences. Nuclear localization signals (NLS), whether classical (monopartite or bipartite) or not, are motifs that direct proteins into the nucleus [25]–[28]. These signals, which are recognized by protein carriers called importins, are characterized by the presence of basic residues, Lys and Arg. In many cases these signals are located near or within other important domains that regulate protein activity [29]. For example, in many transcription factors, NLSs are localized in the proximity of their DNA binding domains [20], [30]. On the other hand, nuclear export signals (NES), which are recognized by exportins and are characterized by hydrophobic amino acids [31], [32], are responsible for the transport of proteins out of the nucleus, back to the cytoplasm. The most common protein involved in exporting cargo from the nucleus is the transporter protein Crm1/Xpo1, first discovered in yeast [33]–[36].

Subcellular localization and protein turnover are two related events that are tightly regulated and control the function of different tumor suppressor proteins. Examples include Rb [37], PTEN [38], BRCA1, p53 and FOXO [39], [40]. Mutations in the corresponding nuclear import-export domains of these proteins disrupt transporter binding, which, in turn, alter their nucleo-cytoplasmic shuttling and, therefore, their normal spatiotemporal dynamics. Among different consequences, protein mislocalization results in abnormal protein turnover and altered function that can promote cell transformation and tumor development [39], [41]–[42].

In this work, we demonstrate that the functional KLF6 NLS is contained within the zinc finger domain but does not include the highly conserved contiguous 5′ basic region (5BR). Moreover, we also identify and characterize a functional NES that regulates KLF6 nucleo-cytoplasmic shuttling in a Crm1-dependent manner. Together, these domains appear to regulate KLF6 nucleo-cytoplasmic transport as well as regulate the half-life of both KLF6 and KLF6-SV1. In sum, these results begin to explain the differences in subcellular localization, half-life and, possibly, function between KLF6 and KLF6-SV1 and how KLF6 gene mutations in these domains and the increase in alternative splicing may result in tumorigenesis.

## Results

### The KLF6 C-terminus zinc finger domain defines nuclear localization

**Figure 2:**
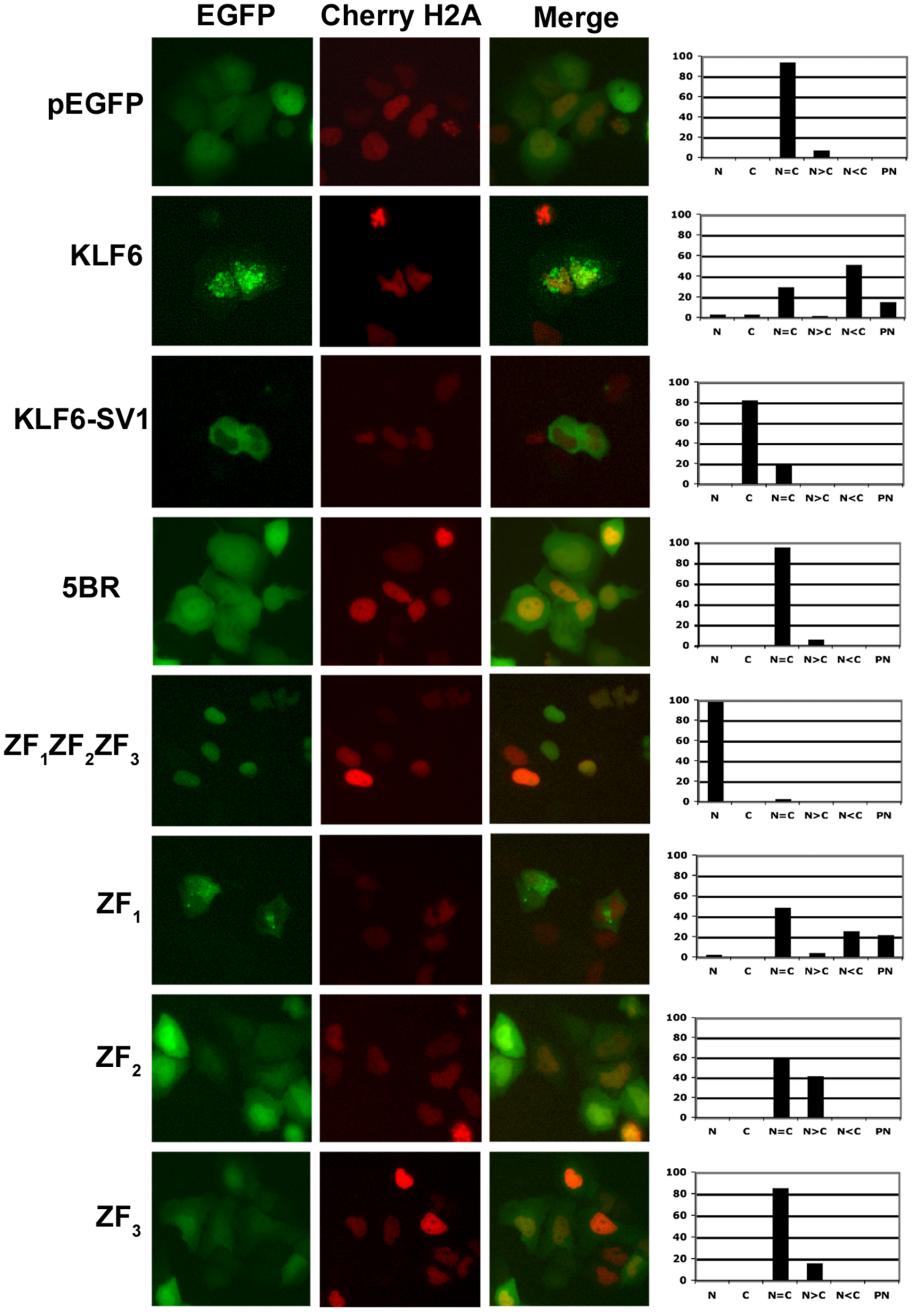
The KLF6 functional NLS resides within the zinc finger domain. Co-localization of KLF6, KLF6-SV1, 5BR or the ZFs EGFP constructs together with Cherry-H2A, which was used to show nuclear staining. Localization of the different constructs was observed by fluorescence microscopy. Graphs with the percentage of cells with the different localization are shown on the right. N, Nuclear localization, C, Cytoplasmic localization, N = C, Nuclear and cytoplasmic distribution within the same cell is equal, N>C, Nuclear localization is more intense than cytoplasmic localization, N<C, Nuclear localization is less intense than cytoplasmic localization, and PN, perinuclear localization.

To investigate whether KLF6 possesses a functional NLS, we generated a series of four constructs encoding truncated KLF6-derived proteins fused to the reporter protein EGFP. The fusion proteins consisted of: pEGFP-KLF6, which encodes the full length KLF6 protein; pEGFP-SV1, full length KLF6-SV1; pEGFP-5BR, the 5′ basic region (5BR); and pEGFP-ZF_1_ZF_2_ZF_3_, the entire KLF6 zinc finger (ZF) domain (Figure 1B). The constructs were transfected into Hela cells and 293T cells and after 24 h their subcellular localization was analyzed by fluorescence microscopy. In agreement with our previous immunohistochemistry findings [16], KLF6 was present both in the nucleus and cytoplasm with areas of intense perinuclear staining, while KLF6-SV1 localized exclusively in the cytoplasm (Figure 2 and Figure S1). The 5′ basic region alone was unable to drive EGFP into the nucleus and cells showed an equal nucleo-cytoplasmic distribution, similar to EGFP control cells. In contrast, cells over-expressing the complete ZF domain had an exclusive nuclear localization pattern (Figure 2 and Figure S1).

To dissect the relative importance of each one of the three ZFs with regard to KLF6 nuclear localization, we engineered three additional constructs wherein each ZF was independently expressed and interrogated: pEGFP-ZF_1_, pEGFP-ZF_2_ and pEGFP-ZF_3_ (Figure 1B). After expressing these constructs in Hela cells, ZF_1_ localization was shown to be similar to that of wild type KLF6 (Figure 2). Namely, ZF_1_ had predominant cytoplasmic staining with some nuclear and perinuclear expression. In contrast, ZF_2_ and ZF_3_ resulted in a more equivalent nucleo-cytoplasmic distribution. Nonetheless, ZF_2_ expressing cells had a more easily distinguishable nuclear staining pattern than ZF_3_ cells (Figure 2).

**Figure 3:**
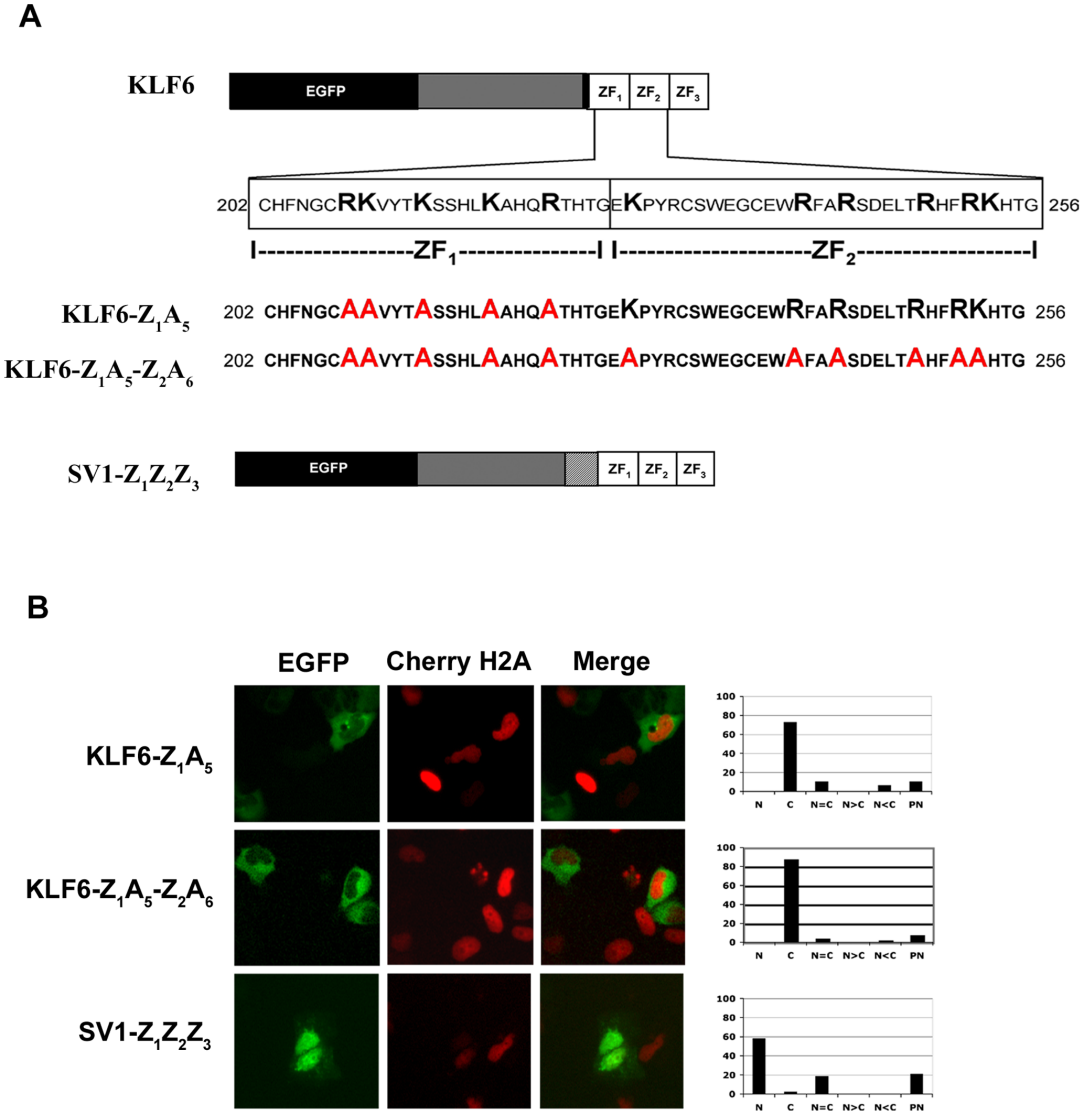
Mutations within the ZFs affect KLF6 nuclear transport. A, Cartoon showing the Ala replacement mutations introduced in ZF_1_ and ZF_2_ and the structure of the chimera SV1-Z_1_Z_2_Z_3_. B, Subcellular localization of the constructs following transfection in Hela cells. Cherry-H2A construct was used to show nuclear staining. Localization of the different constructs was observed by fluorescence microscopy. Graphs with the percentage of cells with the different localization are shown on the right. N, Nuclear localization, C, Cytoplasmic localization, N = C, Nuclear and cytoplasmic distribution within the same cell is equal, N>C, Nuclear localization is more intense than cytoplasmic localization, N<C, Nuclear localization is less intense than cytoplasmic localization, and PN, perinuclear localization.

It has been previously proposed that the basic residues within the ZFs of Krüppel-like factor KLF1 may represent a common NLS motif for all KLF members [21]. It has also been demonstrated that mutations in these basic residues only affect transport but not DNA binding of KLF1 [21]. Given these previous findings, we therefore mutated the basic residues within ZF_1_ and ZF_2_ to more precisely define and map the amino acids involved in KLF6 nuclear localization. Using site-directed mutagenesis we replaced a number of Arg and Lys residues within the ZF domain with Ala (Figure 3A). In total, 11 residues were replaced in both zinc finger domains. The loss of the 5 basic residues in ZF_1_ drastically decreased the number of cells with nuclear and perinuclear localization and increased the number of exclusively cytoplasmically staining cells. Replacement of the 6 basic residues in ZF_2_, along with the altered ZF_1_, further increased the number of cells with cytoplasmic KLF6 localization (Figure 3B). This suggested that in our experimental system, while ZF_1_ may be the main driver of KLF6 nuclear localization, ZF_2_ plays a minor but important role.

Given these findings, one hypothesis that would explain the cytoplasmic localization of KLF6-SV1 is that absence of the ZF domain, and not the 5′ basic region, results in its distinct subcellular localization. To test this, we engineered a KLF6-SV1 construct that possessed all three KLF6 ZFs (KLF6-SV1-Z_1_Z_2_Z_3_). The addition of the complete ZF domain to the chimeric protein resulted in complete re-localization of KLF6-SV1 from the cytoplasm into the nucleus (Figure 3B).

### KLF6 has an NES that is Crm1-dependent

**Figure 4:**
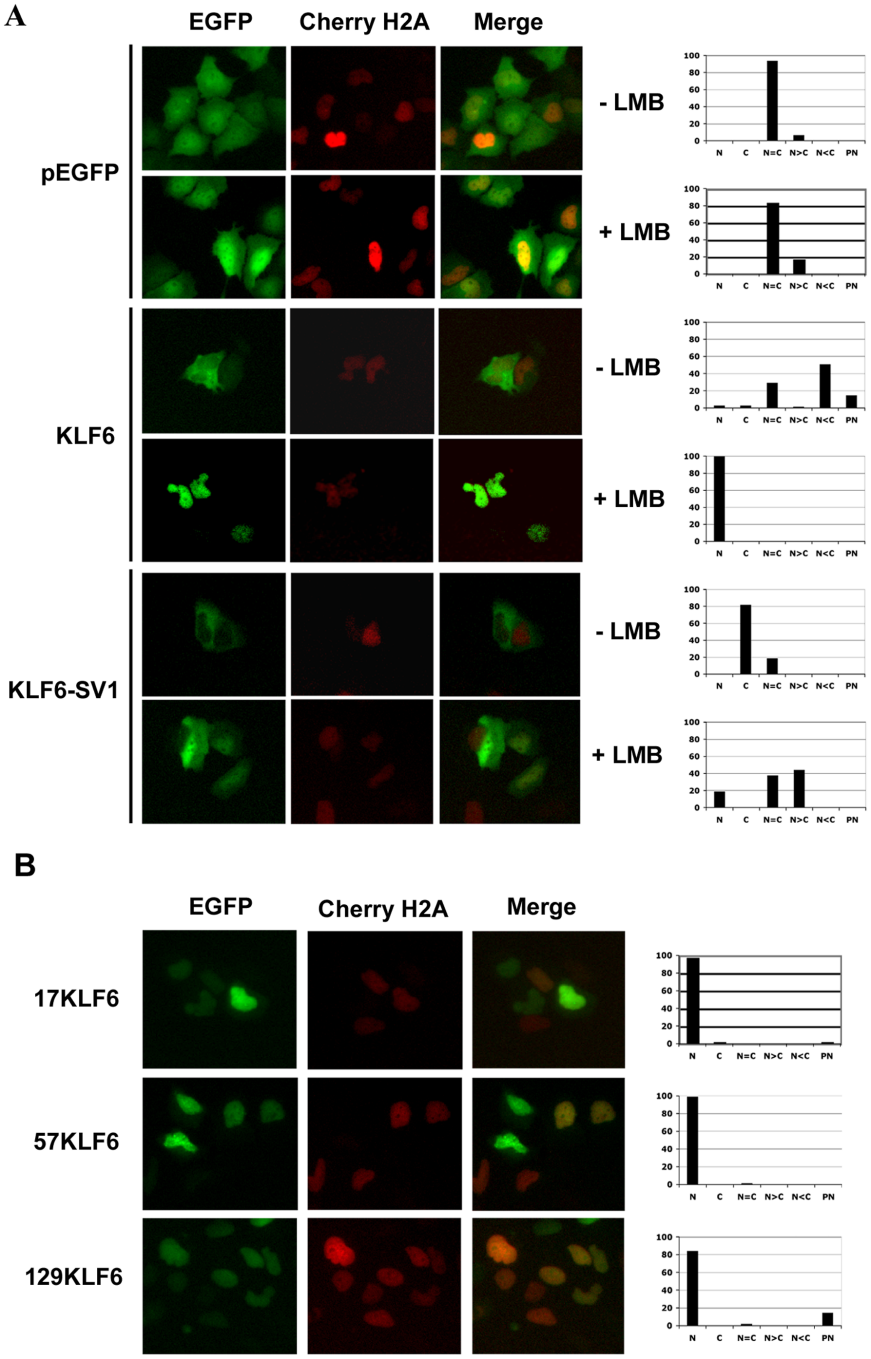
Identification of a Crm1-dependent KLF6/KLF6-SV1 nuclear export signal. A, Hela cells transfected with EGFP-KLF6, EGFP-KLF6-SV1 or empty vector were treated with or without LMB for 2 h. B, The subcellular localization of truncated KLF6 constructs is shown. Cherry-H2A construct was used to show nuclear staining. Localization of the different constructs was observed by fluorescence microscopy. Graphs with the percentage of cells with the different localization are shown on the right. N, Nuclear localization, C, Cytoplasmic localization, N = C, Nuclear and cytoplasmic distribution within the same cell is equal, N>C, Nuclear localization is more intense than cytoplasmic localization, N<C, Nuclear localization is less intense than cytoplasmic localization, and PN, perinuclear localization.

The nucleo-cytoplasmic localization of KLF6 together with the presence of a functional NLS supported the idea that KLF6 could also harbor a functional nuclear export signal (NES). As a first approach to identify this NES, and in order to investigate whether KLF6 nuclear export was mediated by Crm1, we treated Hela cells expressing EGFP-tagged KLF6, KLF6-SV1 or EGFP with the Crm1 inhibitor Leptomycin B (LMB) [43], [44]. In stark contrast to non-LMB treated cells that continued to display both nuclear and cytoplasmic staining of KLF6, LMB treatment resulted in marked KLF6 nuclear accumulation (Figure 4A). Surprisingly, LMB treatment of KLF6-SV1 transfected cells also resulted in nuclear enrichment (Figure 4A). This finding suggests that KLF6-SV1, like the wild type protein KLF6, can also translocate to and exist within the nucleus. We did not explore further whether this was the result of an additional NLS within the primary sequence or possibly through shuttling (“piggy-backing”) with another protein, possibly endogenous KLF6.

For mapping the KLF6 NES domain, we initially used an NES prediction program (http://www.cbs.dtu.dk/services/NetNES/). Using this *in silico* approach only one amino acid with a high score for a putative NES was identified, Ile15. However, manual inspection of the sequence revealed a large number of hydrophobic residues, a common feature of NESs [32], within the first 132 amino acids of the KLF6 protein sequence (highlighted in red in Figure 1A). In order to test whether this region contained a functional NES within this region, we generated three overlapping N-terminus serial deletions and tested their ability to direct transport (Figure 1C). Before microscope visualization, we expressed and analyzed by Western-blot all three truncated proteins (17KLF6, 57KLF6 and 129KLF6), confirming that they were stable and expressed in similar amounts (Data not shown). As displayed in Figure 4B, all three constructs, lacking the first 128 aa, 56 aa and 16 aa, respectively, remained in the nucleus suggesting that all of them lacked a functional NES. Thus, based on the shortest deletion, 16aa, at least one functional NES must exist within this domain and exist within this region.

We then sequentially mutated each of the 9 hydrophobic residues within these first 16 aa to Ala using site-directed mutagenesis (Figure S2). The effect of these mutations on nuclear localization was easily discernible. Only mutations in Val3, Met6, Phe10, Leu13 or Ile15 increased KLF6 nuclear sequestration (Figure S2).

**Figure 5:**
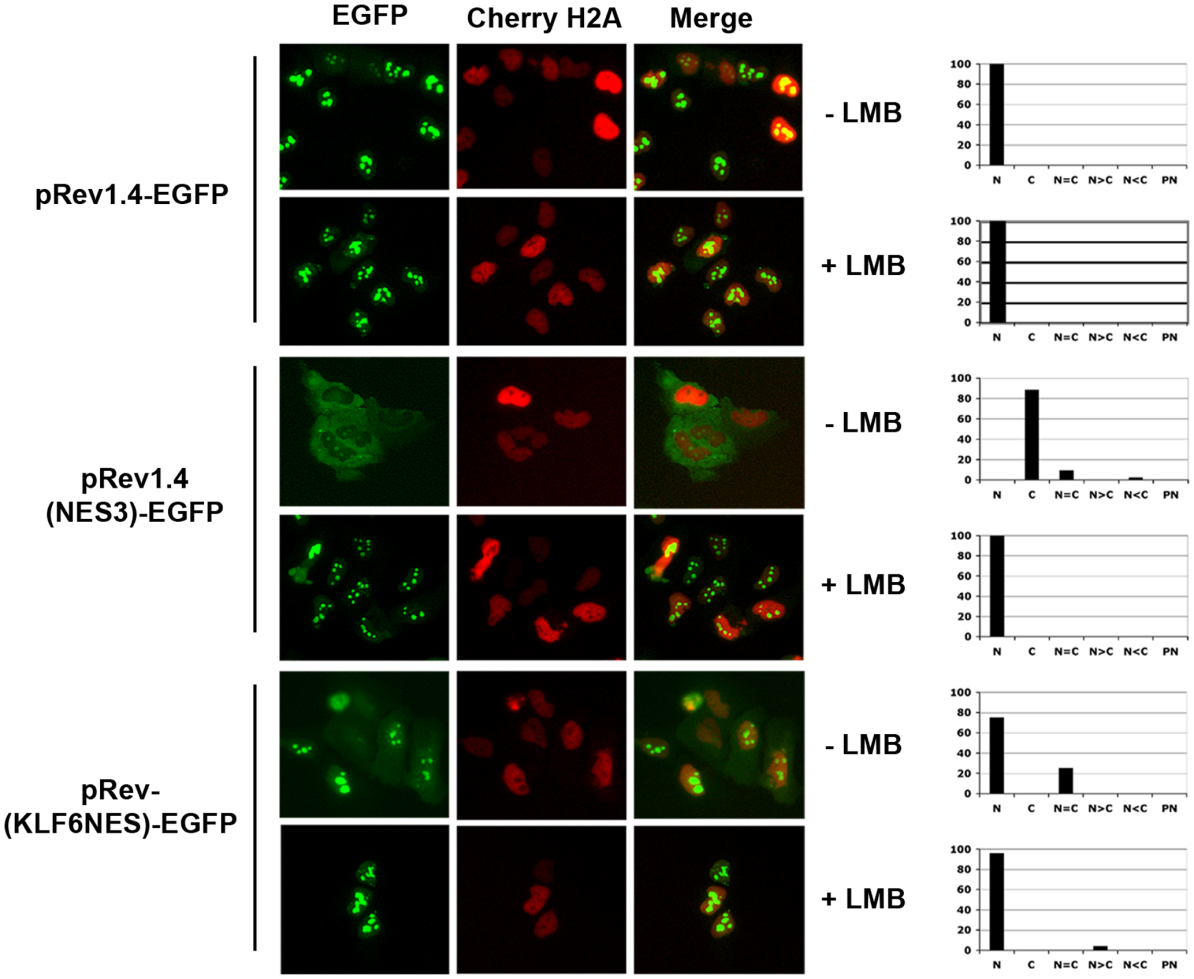
KLF6 presents a CRM1-dependent NES that is of relatively weak strengh. EGFP localization in Hela cells co-transfected with Cherry-H2A and wild type Rev protein (pRev1.4-EGFP), a NLS mutant Rev protein (pRev1.4-(NES3)-EGFP) or a Rev carrying KLF6 NES (pRev-(KLF6NES)-EGFP). Cells were treated or not with LMB for 2 h. Both EGFP and the corresponding fields for Cherry-H2A are shown. Graphs with the percentage of cells with the different localization are shown on the right. N, Nuclear localization, C, Cytoplasmic localization, N = C, Nuclear and cytoplasmic distribution within the same cell is equal, N>C, Nuclear localization is more intense than cytoplasmic localization, N<C, Nuclear localization is less intense than cytoplasmic localization, and PN, perinuclear localization.

The nuclear export rate of a protein depends on the activity of its NES, which in turn is determined by the strength and accessibility to the domain [45]. To gain an approximate understanding of this, we measured the relative strength of the KLF6 NES. Using the system first described by Henderson *et al.*, 2000 we compared the KLF6 NES to that of the human immunodeficiency virus type I (HIV-I) Rev protein. We used three different constructs: pRev1.4-EGFP, encoding an NES-deficient Rev protein; pRev1.4 (NES3)-EGFP, expressing Rev plus its own NES; and pRev1.4 (KLF6 NES)-EGFP that replaces the Rev NES with that from KLF6. Hela cells over-expressing the NES-deficient Rev protein (pRev1.4-EGFP) showed complete nuclear localization whereas the Rev NES containing protein (pRev1.4 (NES3)-EGFP) was exclusively cytoplasmic (Figure 5). However, replacement of the Rev NES with the 16 aa KLF6 NES resulted in partial cytoplasmic relocalization of Rev. Treatment of all 3 transfected cell lines with LMB resulted in complete relocalization of EGFP into the nucleus thus suggesting again the Crm1-dependent nature of the KLF6 NES.

### Nucleo-cytoplasmic transport regulates KLF6 and KLF6-SV1 protein stability

**Figure 6:**
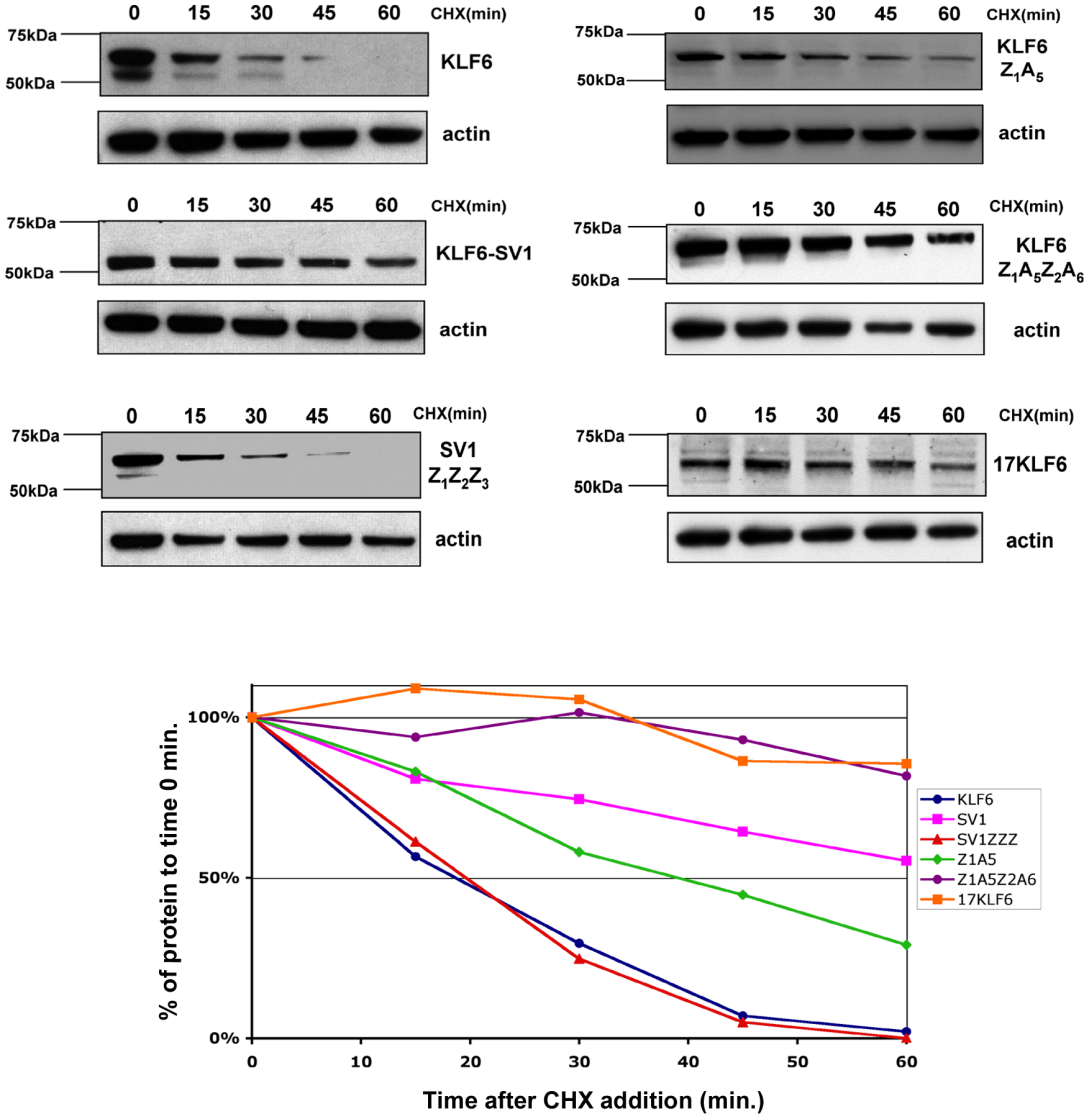
Effects of KLF6 nucleo-cytoplasmic localization domains on protein half-life. Western-blots showing half-life experiments for the wild type and different NLS and NES mutants. Cells were harvested at the times indicated after CHX treatment. Membranes were probed with anti-GFP to detect KLF6, KLF6-SV1 and the mutants, and with anti-actin as a loading control. The graph represents the values obtained after densitometry analysis. The percentage of remaining protein after CHX addition is plotted.

When analyzing their subcellular localization, we noted that cells over-expressing KLF6 showed, in general, less fluorescence compared to those over-expressing KLF6-SV1 or EGFP. Moreover, the different chimeric and mutated KLF6 proteins revealed that fluorescence intensity varied between constructs but not between experiments (data not shown). As protein stability has been linked to protein subcellular localization we investigated whether the half-life of the NLS and NES mutants was different. We treated Hela cells over-expressing the different proteins with cycloheximide (CHX) to inhibit *de novo* protein synthesis and then harvested protein extracts at different time points for Western-blotting. In accord with previous findings [46], wild type KLF6 half-life was ∼18 min (Figure 6).

As predicted, changes in the NLS and NES sequences affected protein stability. The ZF_1_ mutant (KLF6-Z_1_A_5_), doubled KLF6 half-life to ∼40 min. Additional mutations in ZF_2_ (KLF6-Z_1_A_5_Z_2_A_6_) further significantly increased the half-life (Figure 6). Deletion of the NES (17KLF6) also resulted in a markedly increased half-life compared to the wild type protein. The half-life of 17KLF6 was longer than 1 h. Point mutations in one of the mapped critical amino acids (mutant L13AKLF6) were also sufficient to increase KLF6 half-life, having the same effect on half-life as deletion of the complete NES (Data not shown).

KLF6-SV1 has a markedly longer half-life compared to KLF6, >1 h [47]. To determine if KLF6-SV1 stability was also influenced by nucleo-cytoplasmic transport, we added the KLF6 NLS to KLF6-SV1 with the aim of restoring nuclear localization. We generated the chimeric protein SV1-Z_1_Z_2_Z_3_. After transfection in Hela cells, SV1-Z_1_Z_2_Z_3_ restored not only KLF6-SV1 nuclear localization but also resulted in a shorter half-life, ∼19 min, similar to the half-life of the wild type protein KLF6 (Figure 6).

### Nuclear localization affects KLF6 tumor suppressor function

**Figure 7:**
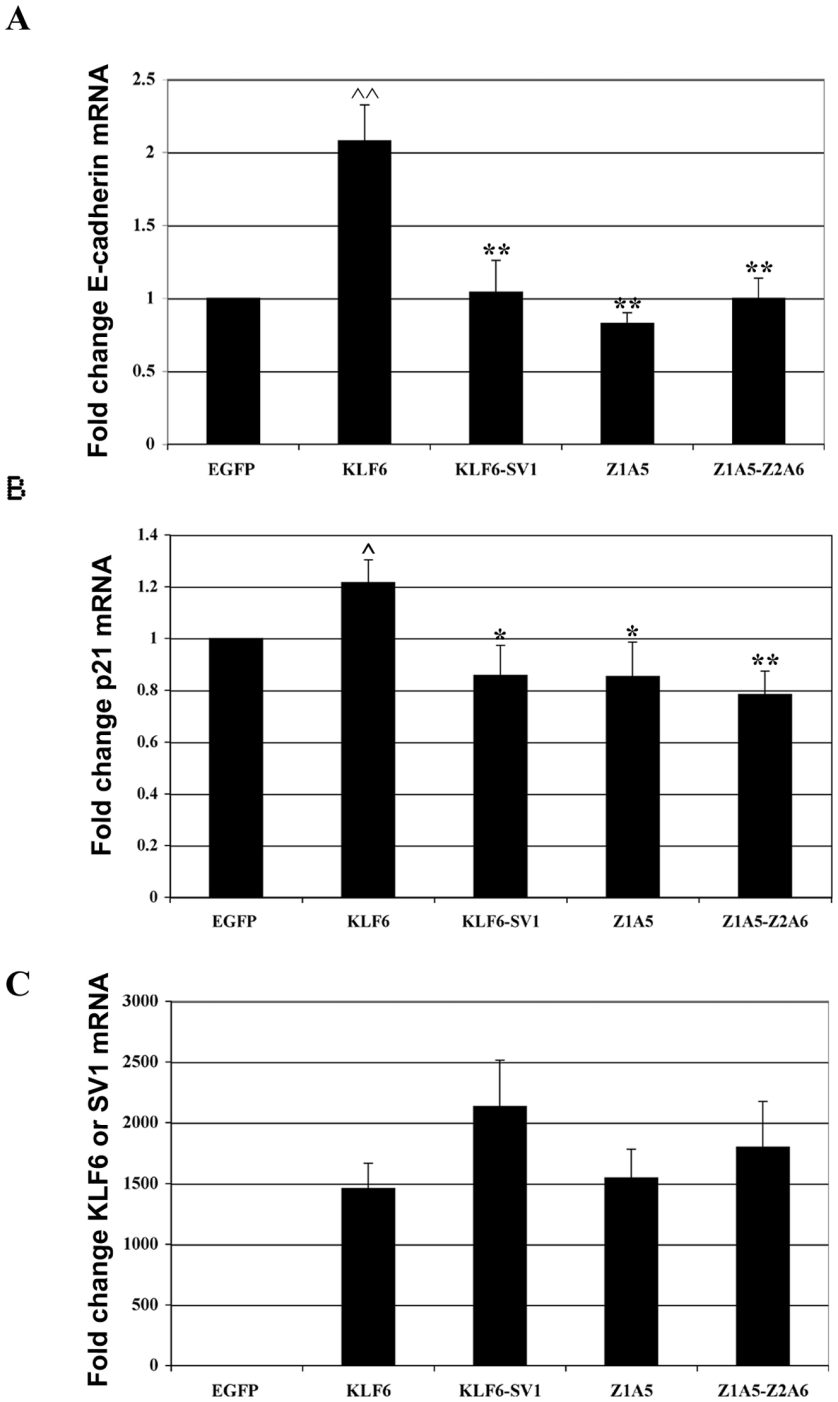
KLF6 intact NLS is necessary for KLF6 tumor suppressor function. RT-PCR data showing endogenous levels of E-cadherin (panel A), p21 (panel B) and the different constructs (panel C). Expression levels were calculated by normalizing each cDNA to GAPDH and then using this normalized value to calculate fold change to the EGFP empty vector value. All experiments were performed at least three times and in triplicate. Statistical significance was determined by two tailed, two-sample equal variance T-test (∧ = p<0.05 and ∧∧ = p<0.005 to EGFP; * =  p<0.05 and ** =  p<0.005 to EGFP-KLF6).

We next investigated whether the differing subcellular localizations of KLF6 and KLF6-SV1 may in part underlie their antagonistic functions. We chose two well-characterized KLF6 transcriptional targets, the transmembrane protein E-cadherin and the cyclin-dependent kinase inhibitor p21. The expression of these two genes has been shown to be increased by wild type KLF6 but not KLF6-SV1 [1], [17], [48]. We used Hela cells over-expressing KLF6, KLF6-SV1 or two NLS mutants (KLF6-Z_1_A_5_, KLF6-Z_1_A_5_Z_2_A_6_) to measure the levels of expression of E-cadherin, by RT-PCR, and p21, by both RT-PCR and luciferase promoter assays. As shown in Figure 7A, cells over-expressing wild type KLF6 doubled E-cadherin expression compared with control vector (p<0.005). Over-expression of KLF6-SV1 or either of the KLF6 NLS mutants had no effect on E-cadherin expression (Figure 7A). Similarly, cells over-expressing KLF6 increased endogenous p21 expression ∼20% (p<0.05) (Figure 7B), and about 4-fold increase when a p21 promoter fused to luciferase gene was used (p<0.005) (Figure S3). No changes were detected in the levels of p21 in cells over-expressing KLF6-SV1 or the two KLF6 NLS mutants in neither one of the experiments. Figure 7C shows the level of expression of the different constructs transfected.

## Discussion

In this work we define and characterize a number of novel regulatory domains and test the mechanisms involved in nucleo-cytoplasmic transport of KLF6 and KLF6-SV1. In turn, these domains seem to be necessary for regulating protein turnover and help to establish functional differences between KLF6 and KLF6-SV1, which have both been shown to play important roles in cancer initiation, progression and survival and for predicting outcome. For example, addition of the KLF6 NLS to KLF6-SV1 results in nuclear localization of this oncogenic protein while markedly decreasing its half-life. Conversely, removing the native NLS sequence from KLF6 resulted in its loss of nuclear targeting but also its inability to activate E-cadherin and p21 transcription.

**Figure 8:**
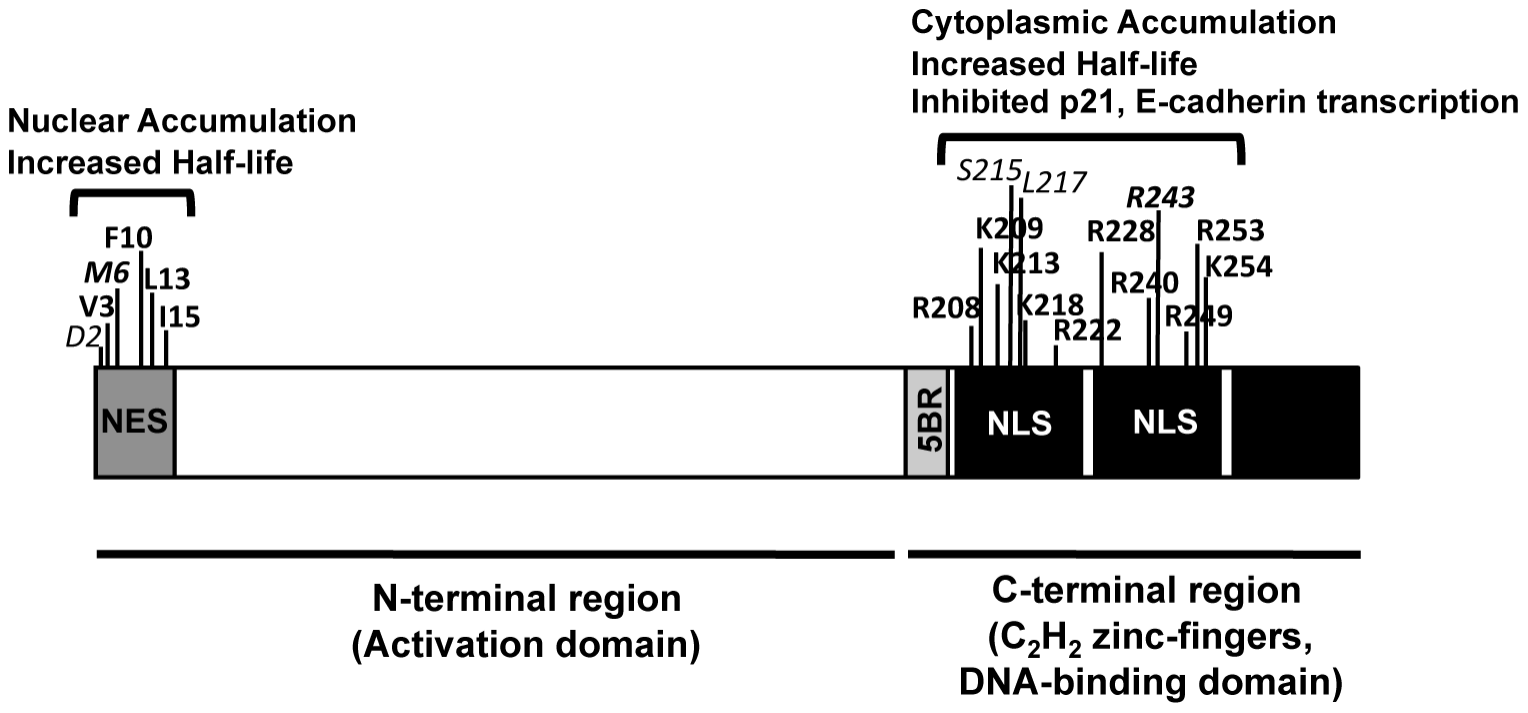
Site-directed and patient-derived mutations in the NES and NLS and their consequences. Site-directed mutations are highlighted in bold, whereas patient-derived mutations, described in the text, are italicized. Overlapping mutations are shown in both bold and italics. For previously published data check reference (55).

KLF6 is frequently inactivated in a number of human cancers. Inactivation can occur through multiple mechanisms including mutation, loss of heterozygosity (LOH), promoter hypermethylation and/or an increase in alternative splicing [1], [5]–[7], [14], [16], [46], [49]. Examination of the published KLF6 mutations demonstrates that indeed a number of the cancer-defined mutations occur in the NLS and NES domains (Figure 8). Three mutations map into the NLS: S215F has been identified in astrocytoma, glioblastoma and meningioma [46], R243K in nasopharyngeal carcinoma [14], and L217S in prostate cancer [1], [5]. In the NES domain, two mutations, D2G and M6V, have also been identified in astrocytoma, glioblastoma and meningioma [50]. Of note, in this study we demonstrated that mutations in amino acids M6 and R243 result in either nuclear localization or cytoplasmic sequestration of KLF6, respectively (Figure 3B, Figure S2 and Figure 8). Given that we demonstrated M6A mutant to have increased nuclear localization, it will be of interest to specifically functionally interrogate the patient-derived KLF6 and KLF6-SV1 M6V mutants, which would both share the mutation, to better understand if their association with cancer arises from loss of the tumor suppressor or activation of the oncogenic variant.

Beyond mutational inactivation, dysregulation of KLF6 alternative splicing has also been described in a number of cancers and increased production of KLF6-SV1 is associated with increased tumor stage [3], chemoresistance [51] and poor prognosis [52]. In one sense, KLF6-SV1 represents a naturally occurring inactivating mutation of the KLF6 NLS. Thus the antagonistic functions of these two proteins can in part be related to their distinct subcellular localizations.

Our demonstration that the KLF6 zinc fingers also encode the functional NLS provides further support for the hypothesis by Pandya *et al.* [21] that a common NLS is present in the zinc finger domain of all KLF family members. Distinctions between the domains do however exist. Different from KLF1 and KLF4 where all three ZFs appear to be necessary for nuclear localization as well as the 5′ basic region adjacent to them [20]–[22], ZF_1_ plays the main role in defining KLF6 subcellular localization (Figure 2). Similar results demonstrating a functional role for ZF_1_ have also been recently demonstrated for KLF8 [23]. On the other hand, ZF_2_, ZF_3_ and the KLF6 5′ basic region sequence (PDGRRRVHR) are not sufficient to direct nuclear localization (Figure 2). These results in deconstructing the functional roles of each zinc finger are in accord with and help to explain previously published findings on the subcellular localization of other KLF6 splice variants. Splice variant 2 (KLF6-SV2), which lacks ZF1 but possesses ZF2 and ZF3, localizes in the cytoplasm [16]. Splice variant 3 (KLF6-SV3), which maintains ZF1 but not ZF2 and ZF3 localizes to the nucleus (Martignetti and Camacho-Vanegas, unpublished results).

Recently, Du *et al.* [24] described the presence of an NES in a KLF family member. The KLF5 NES was shown to be Crm1-dependent and present between aa 119–139 within the regulatory domain and located near a SUMO motif that regulates nuclear export. In this work, we describe that the first 16 amino acids of the common KLF6 and KLF6-SV1 protein sequence contain a NES that might be Crm1-dependent because KLF6 is entrapped in the nucleus following treatment with LMB. Targeted deletions and mutations in some of the hydrophobic residues within this 16 aa domain also resulted in increase in nuclear accumulation. In comparing the strength of the NES to the well-characterized Rev protein, the KLF6 NES was shown to be weaker and thus similar to that of other transcription factors such as p53 and p53-regulated genes like p21 and Hmd2 [45].

One unexpected finding from these studies was the observation that KLF6-SV1, which lacks the KLF6 NLS and which we have previously shown to be localized primarily in the cytoplasm [16] was nonetheless found to be partially relocalized to the nucleus when cells were treated with LMB, a Crm1 inhibitor. This suggests that KLF6-SV1 can be transported into the nucleus in an NLS-independent manner, possibly through binding KLF6 or other actively nucleo-cytoplasmic shuttled proteins (*piggy-backing*). This has been shown to occur with other tumor suppressors including BRCA1, whose NLS-lacking alternatively splice isoforms are transported into the nucleus following DNA damage [53]. In this instance, nuclear transport is mediated through binding to BARD1, another tumor suppressor that heterodimerizes with BRCA1 to form a complex involved in DNA damage repair [53].

Our results link, for the first time, nucleo-cytoplasmic transport of a KLF family member to protein stability. Given KLF6's tumor suppressor function and KLF6-SV1's oncogenic/anti-apoptotic function, this finding may have broad implications. Previous studies showed that KLF6 is ubiquitinated and degraded via the proteasome and has a short half-life of ∼15 min [47]. KLF6-SV1 half-life is appreciably longer [54]. The mechanisms underlying their turnover remained unknown. Here we demonstrate that regulated turnover requires an intact NLS and NES. Disruption of either of them modified KLF6 protein stability. Furthermore, addition of the NLS to KLF6-SV1 not only restored nuclear localization but also decreased protein stability, resulting in a protein with a half-life more similar to wild-type KLF6.

In further agreement with our hypothesis that regulation of nucleo-cytoplasmic transport is a critical determinant of KLF6 function, we demonstrated that mutations in the KLF6 NLS domain result in decreased transcriptional activation of two cancer-relevant targets, p21 and E-cadherin. Access to the nuclear compartment might be a first step of regulation prior to activating target promoters. This has also been demonstrated recently for another KLF member, KLF8, in which the presence of an intact NLS is needed for increased Cyclin D1 transcriptional activation and increased cell proliferation [23]. Ultimately, and given the demonstrated role of KLF6 and KLF6-SV1 in human cancers, it will be important to examine the possible post-traslational modifications which may provide additional layers of regulation to their nucleo-cytoplasmic regulation as well as the mechanism(s) which allow NES-independent KLF6-SV1 nuclear import. The regulation and cellular consequences of nuclear KLF6-SV1 remain to be determined.

## Materials and Methods

### Generation of plasmids and site-directed mutagenesis constructs

The pEGFP-KLF6 plasmid was generated by amplifying the complete KLF6 coding sequence from the pCIneo-KLF6 construct [1] using the primers fwd-KLF6pCIneo and rev-KLF6pCIneo (Table S1). The resulting amplicon was then subcloned using EcoRI sites into the pEGFP-C3 vector (Clontech). The pEGFP-KLF6-SV1 plasmid was generated by cloning the entire KLF6-SV1 coding sequence obtained by EcoRI enzymatic restriction digest from the pCIneo-KLF6-SV1 vector [48] into pEGFP-C3. The pEGFP-5BR construct contains the KLF6 putative NLS sequence (PDGRRRVHR) that was cloned EcoRI/BamHI in pEGFP-C3 from annealing of complementary forward and reverse primers (Table S1). The pEGFP-ZF_1_ZF_2_ZF_3_ construct was made using the primers fwd-Z_1_Z_2_Z_3_ and rev-Z_1_Z_2_Z_3_ (Table S1) to amplify KLF6 zinc fingers (ZF) from the pCIneo-KLF6 vector and then cloned BamHI into the pEGFP-C3 vector. Plasmids pEGFP-ZF_1_, pEGFP-ZF_2_ and pEGFP-ZF_3_, carrying individual KLF6 ZFs, were obtained by cloning KLF6 ZF_1_ (BamHI), ZF_2_ (EcoRI/BamHI), and ZF_3_ (EcoRI/BamHI) sequences amplified by PCR from the pCIneo-KLF6 construct and using the primers fwd-Z_1_Z_2_Z_3_/rev-Z_1_, fwd-Z_2_/rev-Z_2_ and fwd-Z_3_/rev-Z_1_Z_2_Z_3_ (Table S1), respectively. The pEGFP-SV1-Z_1_Z_2_Z_3_ construct was obtained by cloning KLF6-SV1 coding sequence into the pEGFP-ZF_1_ZF_2_ZF_3_ plasmid digested with EcoRI.

We generated the N-terminus deletion constructs lacking the first 128 (pEGFP-129KLF6), 56 (pEGFP-57KLF6) and 16 (pEGFP-17KLF6) amino acids (aa), using the primer combinations fwd-129-283/rev-KLF6pCIneo, fwd-57-283/rev-KLF6pCIneo and fwd-17KLF6/rev-KLF6pCIneo, respectively (Table S1). EcoRI digested amplicons were then subcloned into the pEGFP-C3 vector.

Point mutations in the NES and NLS were sequentially generated in the pEGFP-KLF6 plasmid using commercially available kits following the manufacturer's recommendations (Stratagene, USB Corporation) and the primers listed in Table S2.

The construct pRev-(KLF6NES)-EGFP was made by cloning annealed primers containing the KLF6 NES sequence in the plasmid pRev1.4(NES3)-EGFP digested with BamHI/AgeI. Plasmids pRev1.4 (NES3)-EGFP and pRev1.4-EGFP were kindly donated by Dr. Eric Henderson (Westmead Institute for Cancer Research, Sydney, Australia).

All primer sequences are shown in Table S1 and S2. All expression constructs were confirmed by DNA sequencing in both orientations prior to their use.

### Growth and maintenance of cell lines

All cell lines were purchased from the American Type Culture Collection (Manassas, VA). Cells were grown and maintained in DMEM media (Cellgro®) supplemented with 10% FBS (Gibco) and 1% Penicillin/Streptomycin (Cellgro®). Cells were transfected with Lipofectamine™ 2000 reagent according to the manufacturer's recommendations (Invitrogen).

### Western blot and half-life analysis

Protein extracts for Western blotting were obtained by lysing the cells with radioimmunoprecipitation assay buffer following standard protocols. Protein concentration was measured using the Bio-Rad DC Protein quantification assay and amounts adjusted such that equivalent amounts were loaded (7.5 ug). Electrophoresed proteins were resolved by SDS-PAGE and transferred to a nitrocellulose membrane. Blots were blocked with 5% non-fat milk (Labscientific, Inc.) in TBS-Tween buffer. We used the following primary antibodies: Actin (I-19) (Santa Cruz Biotechnology) and GFP Living Colors (JL-8) (Clontech). Both primary and secondary antibodies were incubated at a dilution of 1:1000 in 5% non-fat milk in TBS-Tween.

For the half-life experiments, Hela cells were transfected with different constructs. The next day, transfected cells were treated with 1 mg/ml of Cycloheximide (Sigma). Protein extracts were obtained at the noted times and then analyzed by Western-blot.

### Fluorescent microscopy

EGFP subcellular localization was observed using a fluorescent microscope (NIKON Eclipse TE 200) with a 20× objective. Photomicrographs were acquired using Spot Advanced Software and the Image J program.

For all localization experiments, two wells of a 6-well plate were transfected and analyzed for each EGFP construct. At the minimum, six [6] fields were randomly chosen and green cells were counted in order to calculate the percentages of nuclear (N), cytoplasmic (C) and perinuclear (PN) cells. In addition, each experiment was repeated at least three times.

### RNA extraction and quantitative real time-PCR (qRT-PCR) analysis

RNA extraction and qRT-PCR analysis were done as previously described [51]. Briefly, RNA was obtained from cells using the Rneasy Mini kit (Qiagen) and treated with DNase (Qiagen). One ug of RNA was used in each reaction to obtain the first-strand complementary DNA by reverse transcription using random primers (Promega). An ABI PRISM 7900HT Sequence Detection System (Applied Biosystems) was used for the qRT-PCR. The primer sequences used have been previously described [3], [16], [48]. All values were normalized to GAPDH levels. All experiments were performed in triplicate and validated thrice independently. Statistical significance was determined by two tailed, two-sample equal variance *T*-test (∧ =  p<0.05 and ∧∧ = p<0.005 to EGFP; * =  p<0.05 and ** =  p<0.005 to EGFP-KLF6).

### Luciferase transactivation assays

Hela cells transfected with a p21 promoter construct (1 ug) and either KLF6, KLF6-SV1, the NLS mutants or EGFP empty vector (1 ug) were harvested 24 h after transfection. Dual-Luciferase® Reporter Assay kit (Promega, Madison, WI, USA) was used to extract protein and develop the assay following the manufacturer's recommendations. The TK promoter-Renilla Luciferase construct (Promega, Madison, WI, USA), 10 ng, was used to normalize each experiment. Luciferase activity was determined for each EGFP construct by luminescence in a ModulusTM II Microplate Multimode Reader (Promega, Madison, WI, USA). All experiments were performed in triplicate and validated thrice independently. Statistical significance was determined by two tailed, two-sample equal variance T-test (p<0.005).

## Supporting Information

Figure S1 Localization of KLF6, KLF6-SV1, KLF6 NLS and the ZFs in 293T cells. Co-localization of KLF6, KLF6-SV1, KLF6 NLS or the ZFs EGFP constructs together with Cherry-H2A, which was used to show nuclear staining. Localization of the different constructs was observed by fluorescence microscopy.
(1.17 MB TIF)

Figure S2 Mutations in the N-terminus 16 amino acids results in increased KLF6 nuclear localization. Subcellular localization of the different NES mutants. Cherry-H2A construct was used to show nuclear staining. Localization of the different constructs was observed by fluorescence microscopy. Graphs with the percentage of cells with the different localization are shown on the right. N, Nuclear localization, C, Cytoplasmic localization, N = C, Nuclear and cytoplasmic distribution within the same cell is equal, N>C, Nuclear localization is more intense than cytoplasmic localization, N<C, Nuclear localization is less intense than cytoplasmic localization, and PN, perinuclear localization.
(1.62 MB TIF)

Figure S3 p21 promoter luciferase assays for KLF6, KLF6-SV1 and the NLS mutants as well as EGFP empty vector in 293T cells. Expression levels were calculated by normalizing each luciferase value to Renilla gene expression and representing the Relative Luciferase Units (RLU). All experiments were performed at least three times and in triplicate. Statistical significance was determined by two tailed, two-sample equal variance T-test (p<0.005).
(0.08 MB TIF)

Table S1 Primers used for site-directed mutagenesis. ‘P’ represents the primers that are 5′ phosphorylated.
(0.04 MB DOC)

Table S2 Primers used to generate expression constructs. Restriction sites are underlined.
(0.04 MB DOC)
