# Supplementary figures and images for: Nucleo-Cytoplasmic Localization Domains Regulate Krüppel-Like Factor 6 (KLF6) Protein Stability and Tumor Suppressor Function
Source: PLoS One. 2010 Sep 9;5(9):e12639. doi: 10.1371/journal.pone.0012639 (PMC2936564; doi:10.1371/journal.pone.0012639)

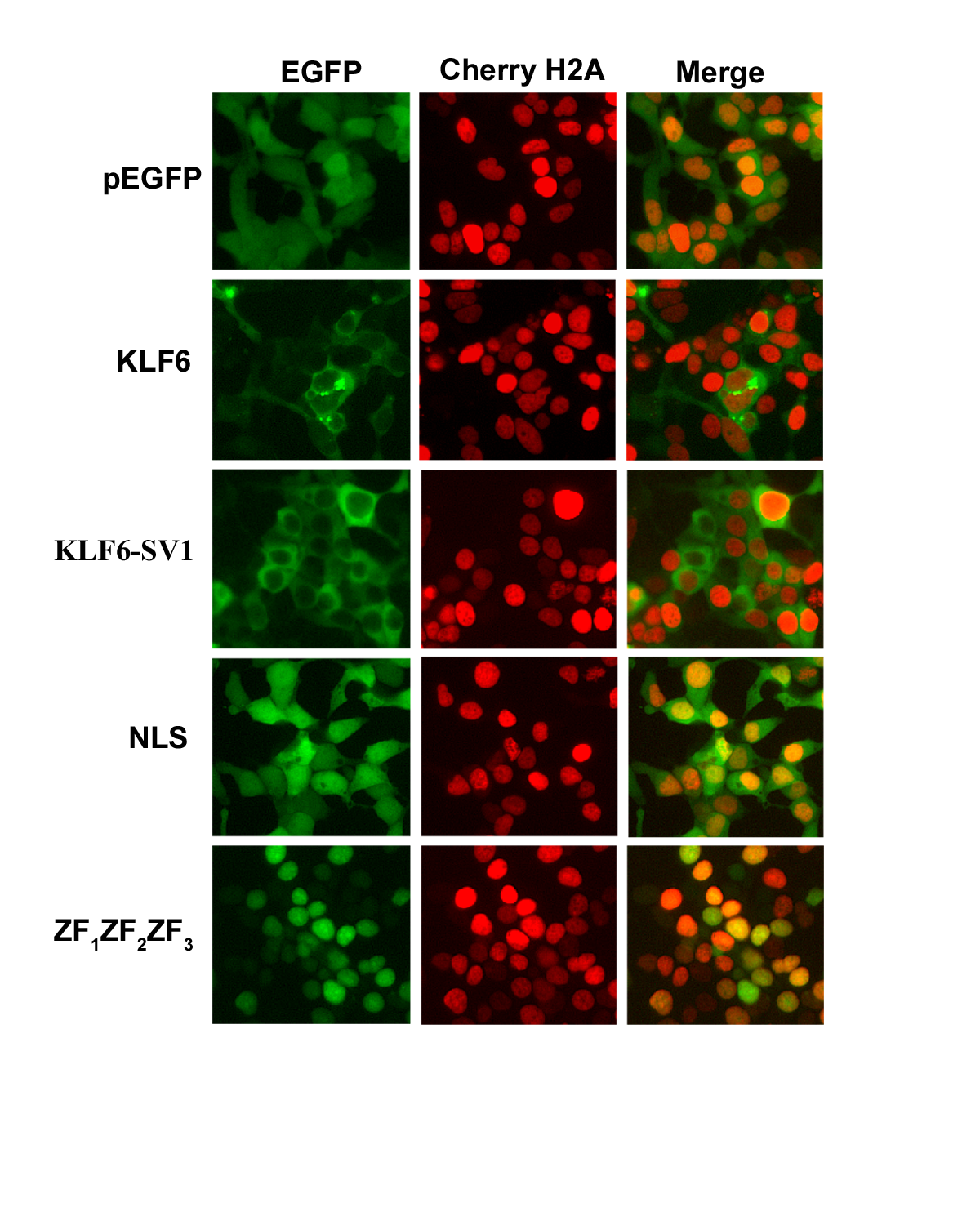

Supplement: Figure S1 — Localization of KLF6, KLF6-SV1, KLF6 NLS and the ZFs in 293T cells. Co-localization of KLF6, KLF6-SV1, KLF6 NLS or the ZFs EGFP constructs together with Cherry-H2A, which was used to show nuclear staining. Localization of the different constructs was observed by fluorescence microscopy. (1.17 MB TIF) [file pone.0012639.s001.tif]

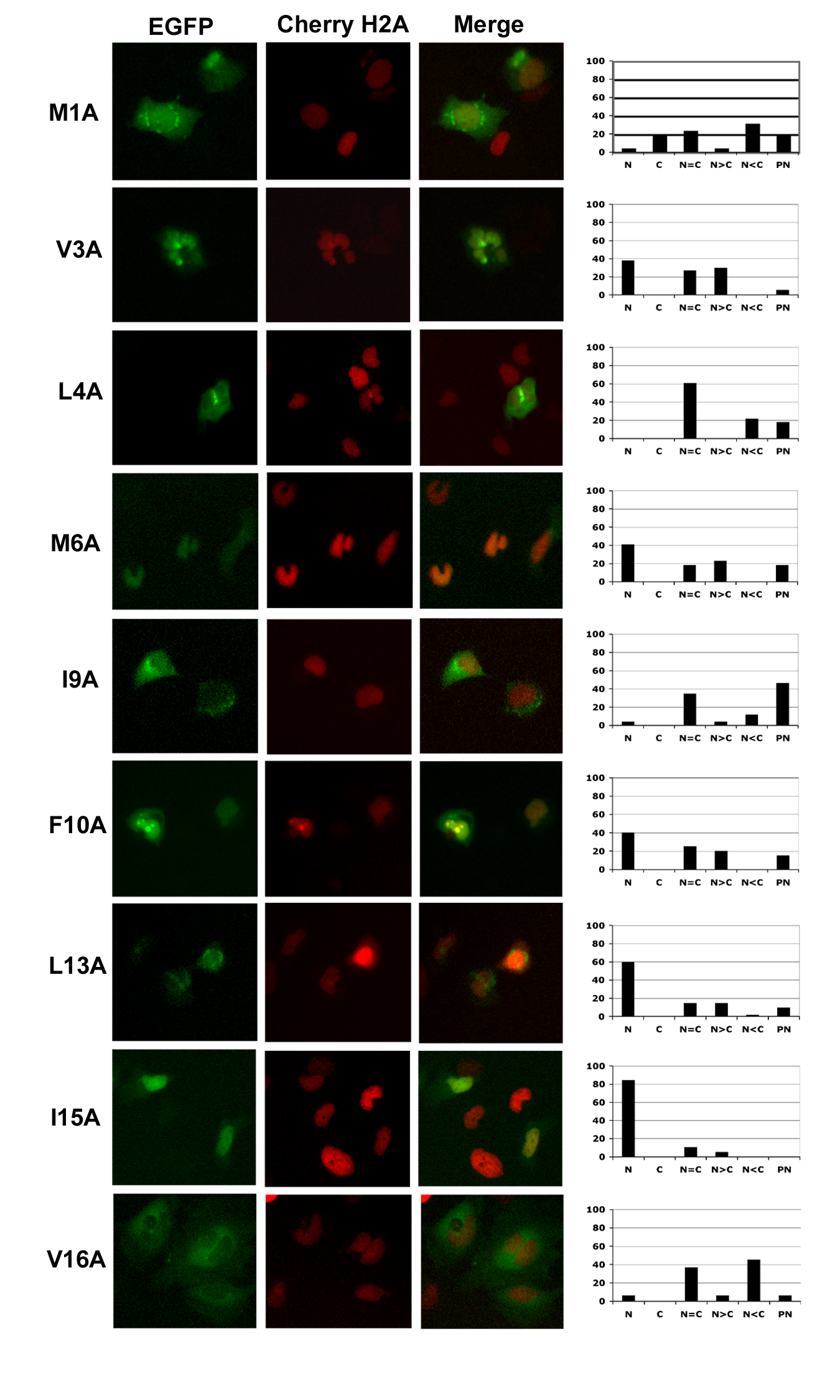

Supplement: Figure S2 — Mutations in the N-terminus 16 amino acids results in increased KLF6 nuclear localization. Subcellular localization of the different NES mutants. Cherry-H2A construct was used to show nuclear staining. Localization of the different constructs was observed by fluorescence microscopy. Graphs with the percentage of cells with the different localization are shown on the right. N, Nuclear localization, C, Cytoplasmic localization, N = C, Nuclear and cytoplasmic distribution within the same cell is equal, N>C, Nuclear localization is more intense than cytoplasmic localization, N<C, Nuclear localization is less intense than cytoplasmic localization, and PN, perinuclear localization. (1.62 MB TIF) [file pone.0012639.s002.tif]

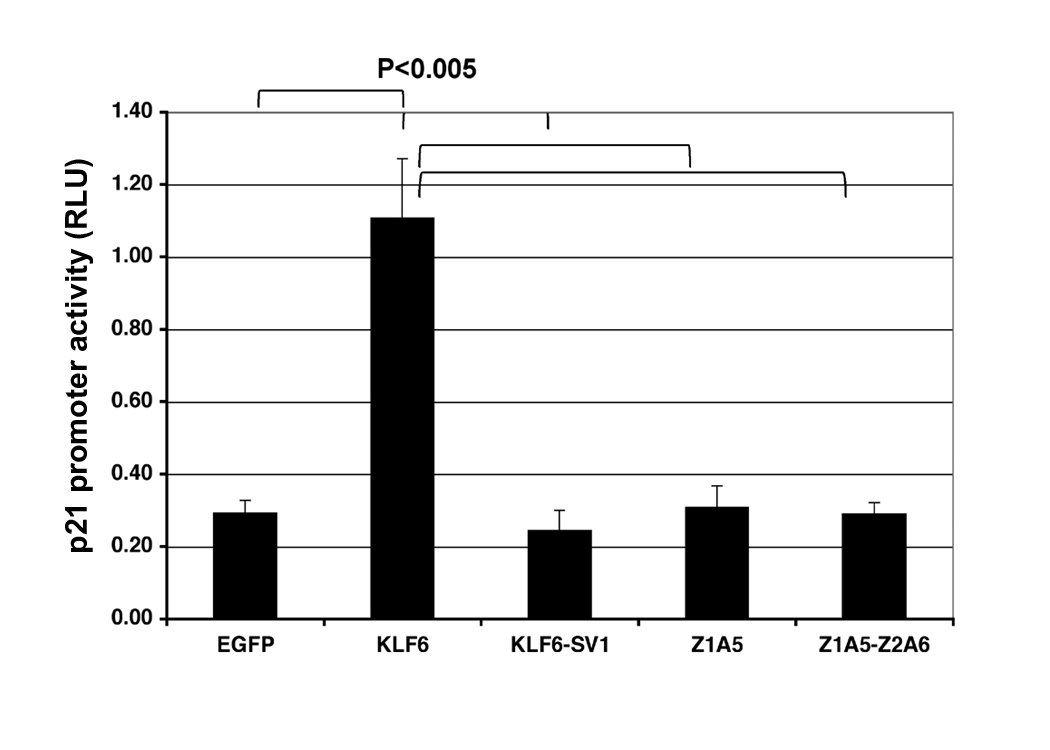

Supplement: Figure S3 — p21 promoter luciferase assays for KLF6, KLF6-SV1 and the NLS mutants as well as EGFP empty vector in 293T cells. Expression levels were calculated by normalizing each luciferase value to Renilla gene expression and representing the Relative Luciferase Units (RLU). All experiments were performed at least three times and in triplicate. Statistical significance was determined by two tailed, two-sample equal variance T-test (p<0.005). (0.08 MB TIF) [file pone.0012639.s003.tif]
