# Supplementary material for: Nucleo-Cytoplasmic Localization Domains Regulate Krüppel-Like Factor 6 (KLF6) Protein Stability and Tumor Suppressor Function
Source: PLoS One. 2010 Sep 9;5(9):e12639. doi: 10.1371/journal.pone.0012639 (PMC2936564; doi:10.1371/journal.pone.0012639)
Supplement: Table S1 — Primers used for site-directed mutagenesis. ‘P’ represents the primers that are 5′ phosphorylated. (0.04 MB DOC) [file pone.0012639.s004.doc]

| **PRIMER NAME** | SEQUENCE 5’-3’ |
| --- | --- |
| fwdKLF6pCIneo | GAATTCCCGACATGGACGTGCTC |
| revKLF6pCIneo | GAATTCCCACCTCTTTGCTCCCTCA |
| fwdNLS2 | AATTCCCGACGGCAGGAGGAGGGTGCACCGG |
| revNLS2 | GATCCCGGTGCACCCTCCTCCTGCCGTCGGG |
| fwdKLF6Z_1_Z_2_Z_3_ | GGATCCACACCGGTGCCACTTTAAC |
| revKLF6Z1Z2Z3 | GGATCCTCAGAGGTGCCTCTTCATG |
| revZ_1_ | GGATCCTGTGTGCGTCCGCTG |
| fwdZ_2_ | GAATTCGAGAAAAGCCTTACAGATGCTCATGG |
| revZ_2_ | GGATCCCGGTGTGCTTTCGGAAG |
| fwdZ_3_ | GAATTCCCGCCAAGCCTTTTAAATGCTC |
| fwd129-283 | GAATTCGGCCCATTGGCGAAGTTTTGG |
| fwd57-283 | GAATTCGGTTTGACAGCCAGGAAGATCTG |
| fwd17KLF6 | GAATTCACGAGACCGGCTACTTCTCG |
| fwd16aaKLF6NES | GATCCAATGGACGTGCTCCCCATGTGCAGCATCTTCCAGGAGCTCCAGATCGTGCCA |
| rev16aaKLF6NES | CCGGTGGCACGATCTGGAGCTCCTGGAAGATGCTGCACATGGGGAGCACGTCCATTG |
