# Supplementary material for: Nucleo-Cytoplasmic Localization Domains Regulate Krüppel-Like Factor 6 (KLF6) Protein Stability and Tumor Suppressor Function
Source: PLoS One. 2010 Sep 9;5(9):e12639. doi: 10.1371/journal.pone.0012639 (PMC2936564; doi:10.1371/journal.pone.0012639)
Supplement: Table S2 — Primers used to generate expression constructs. Restriction sites are underlined. (0.04 MB DOC) [file pone.0012639.s005.doc]

| **PRIMER NAME** | **SEQUENCE 5’-3’** |
| --- | --- |
| fwdM1A | P-GAATTCCCGACGCGGACGTGCTCC |
| fwdV3A | P-CGACATGGACGCGCTCCCCATG |
| fwdL4A | ACATGGACGTGGCCCCCATGTGCAG |
| revL4A | CTGCACATGGGGGCCACGTCCATGT |
| fwdI9A | P-CATGTGCAGCGCCTTCCAGGAG |
| fwdF10A | P-GTGCAGCATCGGCCAGGAGCTC |
| fwdL13A | TCTTCCAGGAGGCCCAGATCGTGCA |
| revL13A | TGCACGATCTGGGCCTCCTGGAAGA |
| fwdI15A | AGGAGCTCCAGGCCGTGCACGAGAC |
| revI15A | GTCTCGTGCACGGCCTGGAGCTCCT |
| fwdV16A | P-GCTCCAGATCGCGCACGAGACC |
| fwdR208AK209A | P-AACGGCTGCGCGGCAGTTTACACC |
| fwdK213A | P-GCAGTTTACACCGCAAGCTCCCACTTG |
| revK218A | P-GCTGGTGTGCTGCCAAGTGGGAGC |
| revR222A | P-CTGTGTGCGTCGCCTGGTGTGC |
| fwdK228A | P-CACACAGGAGAAGCGCCTTACAGATG |
| fwdR240A | P-GGTGTGAGTGGGCTTTTGCAAGAA |
| fwdR243A | P-GGGCTTTTGCAGCAAGTGATGAGT |
| revR249A | P-TCGGAAGTGCGCGGTTAACTCA |
| revR252AK253A | P-CGGTGTGCGCTGCGAAGTGCG |
| Amp REV | P-GTCAGAAGTAAGTTGGCCGCAGTGTTATCACTCATGG |
| revKANA | P-GGTAGCCGGATCAAGCGTATGCAGC |
